# Supplementary material for: Perfectionistic Children and Their Parents: Is There Room for an Intergenerational Transmission? A Study of a Clinical Sample of Italian Children and Their Parents
Source: Children (Basel). 2023 Feb 26;10(3):460. doi: 10.3390/children10030460 (PMC10047207; doi:10.3390/children10030460)
Supplement: Supplementary file 1 [file children-10-00460-s001.zip › children-2193217-supplementary.pdf]

Supplementary Materials

Table S1. Pearson correlations between parent MPS-S and children CAPS dimensions

Variable	MPS-SOP	MPS-OOP	MPS-SPP
SOP-Striving	,255**	,214*	,315**
SOP-Critical	,252*	,320**	,410**
CAPS-SPP	,175	,231*	,272**

Note. **. p < .01; *. p < .05

Table S2. Pearson inter-correlations CAPS dimensions

Variable	SOP-Striving	SOP-Critical	CAPS_SPP
SOP-Striving	1	,607**	,321**
SOP-Critical	,607**	1	,483**
CAPS-SPP	,321**	,483**	1

Note. **. p < .01; *. p < .05

Table S3. Pearson inter-correlations between MPS-S subscales

Variable	MPS-SOP	MPS-OOP	MPS-SPP
MPS-SOP	1	,697**	,640**
MPS-OOP	,697**	1	,660**
MPS-SPP	,640**	,660**	1

Note. **. p < .01; *. p < .05

Table S4. Pearson correlations between CAPS subscales and HiPIC dimensions

Variable	SOP-Striving	SOP-Critical	CAPS-SPP
Anxiety	,265**	,225*	,050
Self-confidence	,117	-,108	-,124
Energy	,076	,044	,051
Expressiveness	,009	,110	,026
Optimism	,093	,022	-,072
Shyness	,230*	,166	,071
Creativity	,234*	,206*	,069
Intellect	,318**	,094	-,123
Curiosity	,083	,073	-,134
Altruism	,219*	-,033	-,067
Dominance	-,034	,266**	,124
Egocentrism	,068	,262**	,248*
Compliance	,086	,115	-,013
Irritability	,023	,051	,030
Concentration	,152	,094	,008
Perseverance	,059	,012	-,018
Order	-,020	,147	,015
Achievement striving	,203*	,312**	-,059
Emotional Stability	-,106	-,071	,081
Extraversion	-,062	,042	-,008
Imagination	,290**	,179	-,088
Benevolence	,239*	-,009	-,072
Cconscientiousness	,193	,259**	-,065

Note. **. $p < .01$; *. $p < .05$
